# Rapid Assessment of Ecosystem Services Provided by Two Mineral Extraction Sites Restored for Nature Conservation in an Agricultural Landscape in Eastern England

**DOI:** 10.1371/journal.pone.0121010

**Published:** 2015-04-20

**Authors:** Phillip J. Blaen, Li Jia, Kelvin S.-H. Peh, Rob H. Field, Andrew Balmford, Michael A. MacDonald, Richard B. Bradbury

**Affiliations:** 1 RSPB Centre for Conservation Science, RSPB, The Lodge, Sandy, Bedfordshire, SG19 2DL, United Kingdom; 2 School of Geography, Earth and Environmental Sciences, University of Birmingham, Edgbaston, Birmingham, B15 2TT, United Kingdom; 3 School of Biological Sciences, University of Hong Kong, Pokfulam, Hong Kong, People’s Republic of China; 4 Conservation Science Group, Department of Zoology, University of Cambridge, Downing Street, Cambridge, CB2 3EJ, United Kingdom; 5 Institute for Life Sciences, University of Southampton, University Road, Southampton, SO17 1BJ, United Kingdom; Chinese Academy of Sciences, CHINA

## Abstract

Despite growing recognition that mineral sites restored for nature conservation can enhance local biodiversity, the wider societal benefits provided by this type of restoration relative to alternative options are not well understood. This study addresses this research gap by quantifying differences in ecosystem services provision under two common mineral site after-uses: nature conservation and agriculture. Using a combination of site-specific primary field data, benefits transfer and modelling, we show that for our sites restoration for nature conservation provides a more diverse array of ecosystem services than would be delivered under an agricultural restoration scenario. We also explore the effects of addressing different conservation targets, which we find alter the provision of ecosystem services on a service-specific basis. Highly species-focused intervention areas are associated with increased carbon storage and livestock grazing provision, whereas non-intervention areas are important for carbon sequestration, fishing, recreation and flood risk mitigation. The results of this study highlight the wider societal importance of restored mineral sites and may help conservation managers and planners to develop future restoration strategies that provide benefits for both biodiversity and human well-being.

## Introduction

Mineral extraction is an important economic industry, with sand and gravel excavation in the UK alone valued at £665m a^-1^ [1]. Extraction activities are transient, ranging from less than a year to several decades in duration, and once completed mineral companies are often required to implement a restoration management plan to transform sites to an appropriate after-use. Among the many potential options, restoration to agricultural land is prevalent in the UK, accounting for over half of all restored sites since the mid-20^th^ Century [2]. However, with increasing recognition of the potential of former mineral sites to enhance local biodiversity [3–5], the proportion of post-extraction sites that are or will be restored to nature conservation areas is increasing, and now accounts for approximately 30% of all sites [6].

Nature conservation is mostly promoted and implemented on the grounds of the intrinsic value of biodiversity, yet conservation areas can also provide multiple ecosystem service benefits such as water quality improvement or carbon storage and sequestration [7–10]. However, while the biodiversity potential of mineral sites restored for nature conservation is well-recognised, their capacity to deliver wider societal benefits is less understood (but see [11]). With growing concerns around food security, competing demands for land [12, 13], and the costs associated with nature conservation [14], there is a need to evaluate the ecosystem services provided by mineral sites restored for nature conservation relative to those restored for agriculture. Furthermore, recent work has suggested ecosystem service delivery at conservation sites may vary depending on the extent to which management activities focus on protecting particular species and habitats through targeted interventions [15]. However, there is currently minimal knowledge regarding how the degree of species-focused intervention in nature conservation affects ecosystem service provision from restored mineral sites [16].

In this study, we begin to address these research gaps by using a rapid assessment toolkit to investigate ecosystem services provided by two restored mineral extraction sites in a predominantly agricultural landscape in Cambridgeshire, UK. Our objectives were to: (1) quantify differences in ecosystem service provision between mineral sites restored for agriculture and nature conservation; and (2) understand the extent to which the degree of species-focused intervention in nature conservation affects ecosystem service provision at restored mineral sites.

## Methods

### Study sites

**Fig 1:**
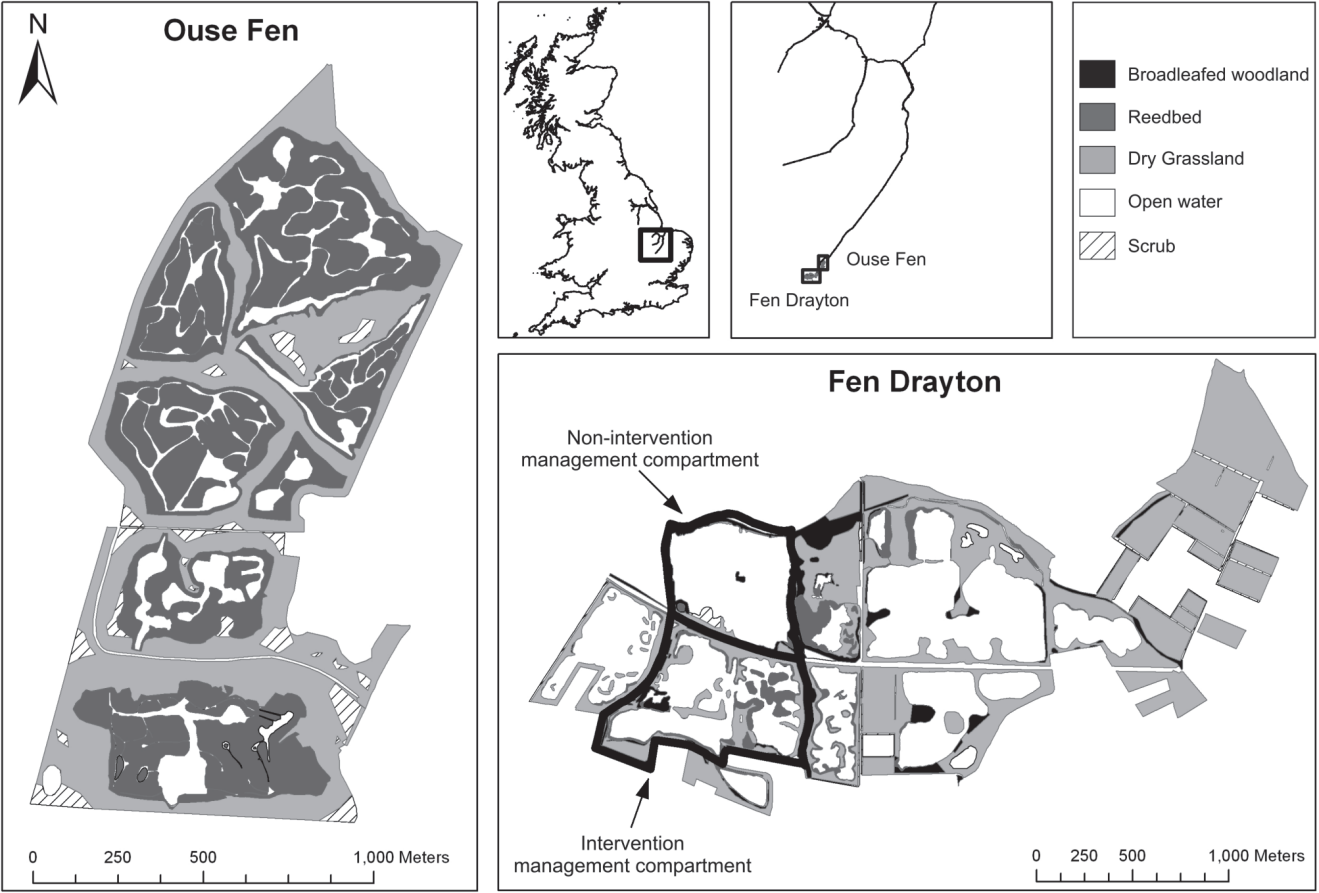
Map of Ouse Fen and Fen Drayton showing main habitat types present at each site. Note that broadleaved woodland and scrub areas are combined for estimates of global climate change mitigation.

The study was conducted at two former gravel extraction sites: Ouse Fen Nature Reserve (otherwise known as the Hanson-RSPB wetland project; 52.33 N, 0.02 W) and Fen Drayton Lakes Nature Reserve (52.30 N, 0.04 W), located in Cambridgeshire, UK (Fig. 1). Both sites are managed by the Royal Society for the Protection of Birds (RSPB) and permission for conducting research was obtained from site managers prior to commencement of fieldwork activities. The sites are surrounded by arable farmland and are recognised as important birdwatching locations. The population of the immediate surrounding area is relatively low; the nearest population centres to both sites are Huntingdon, approximately 12 km away with a population of 24,000, and Cambridge, approximately 20 km away with a population of 124,000 [17].

### Ouse Fen Nature Reserve

The site of Ouse Fen Nature Reserve has been used by Hanson UK for gravel extraction since 1994. Approval for mineral extraction was originally granted based on an intended after-use of agriculture. However, this plan was later changed to a nature-conservation after-use scheme, and restoration began in 2002. Nature-focused restoration includes extensive profiling of the post-extraction basin to create shallow areas, and deliberate planting with reeds. At the time of study, the post-extraction nature reserve site was 153 ha. When extraction is completed in 2032, it is expected that the resulting reserve will be about 700 ha. The site contains four primary habitat types: (i) open water and (ii) *Phragmites australis* reedbed in the post-extraction basins; and (iii) grassland and (iv) scrub woodland (predominantly *Crataegus monogyna* and *Prunus spinosa*) in the areas between these basins. Despite its short history as a nature reserve, Ouse Fen has already been colonised by several bird species of high conservation value including bittern (*Botaurus stellaris*), marsh harrier (*Circus aeruginosus*) and bearded reedling (*Panurus biarmicus*) [18]. Cattle grazing is used to manage the extensive grassland swards and a network of paths is being created to provide visitor access from several entry points.

### Fen Drayton Lakes Nature Reserve

Fen Drayton Lakes Nature Reserve is a 311 ha site situated approximately 3 km southwest of Ouse Fen. Like Ouse Fen, the site comprises a series of lakes, wet grassland, scrub woodland and *P*. *australis* reedbed, in this case formed progressively as a result of gravel extraction between 1950 and 1997. Initially, parts of the site that had been extracted received minimal human intervention, resulting in deep basins with fringing vegetation that was allowed to colonise naturally. We refer to this as our non-intervention scenario. Parts that were extracted later received restoration that was more nature-focused, including profiling basins to provide shallow areas for reed-bed development, with a particular emphasis on attracting target species of conservation concern. We refer to this as our intervention scenario. The site therefore consists of a mix of non-intervention (older) and intervention (newer) compartments. The RSPB acquired the site in 2007 and have implemented biodiversity-sensitive management within and between compartments, including grazing by cattle and sheep to maintain wet grassland areas. An extensive series of paths between compartments provides for visitor access from a series of entry points.

### Ecosystem service assessment and scenario development

Discussions with key stakeholders—including RSPB reserve managers, the Environment Agency, regulators, and business partners—were used to identify the key ecosystem services provided by each site in their current state and under plausible alternative land use or management scenarios. To address Objective 1, ecosystem service delivery at Ouse Fen was compared between the following post-extraction land use scenarios:

- wetland nature conservation, as per the current state and extent of the site
- agriculture, as per the original restoration proposal

To address Objective 2, ecosystem service delivery at Fen Drayton was compared under the following land use scenarios:

- current state of the site as a whole, with a mixture of invention and non-intervention compartments;
- intervention scenario, focusing on targeted species-focused restoration and management actions across the entire site;
- non-intervention scenario, without profiling of the post-extraction basins and allowing natural vegetation colonisation across the entire site.

The key ecosystem services provided at Ouse Fen were considered by stakeholders to be global climate change mitigation (through carbon storage and sequestration) and nature-based recreation (under both scenarios), crop production (in the agricultural scenario), and grazing (under the current nature reserve scenario). At Fen Drayton, the key ecosystem services were identified as global climate change mitigation, livestock grazing, fishing, nature-based recreation and flood risk mitigation (provided by all scenarios). Ecosystem services were assessed for the current and alternative restoration scenarios at each site using the TESSA (Toolkit for Ecosystem Service Site-based Assessment) methodology [19] to quantify differences in ecosystem service delivery attributable to a given restoration strategy. TESSA is designed to provide practical, affordable and accessible methods for quantifying how the net value of ecosystem services at the site scale is likely to change under different management decisions. This approach was chosen because most other available assessment ‘toolkits’ are either designed primarily for use at larger spatial scales, rely heavily on existing secondary datasets, or are unable to assess relative differences in service provision under different scenarios [19].The following sub-sections describe assessment methods for each of these ecosystem services.

### Global climate change mitigation

Current maps of Ouse Fen and Fen Drayton were used to stratify each site into four main habitat types: grassland, scrub woodland, *Phragmites australis*-dominated reedbed and open water [Table 1]. For the agriculture scenario at Ouse Fen, it was assumed that the site would be returned to arable farmland typical of the area surrounding the site with a regionally-characteristic mixture of cereal and general cropping. Using figures from the June agricultural census in England [20] for Cambridgeshire in 2010, we assumed a ratio of 63% cereals and 37% general cropping. We used the three dominant cereal (winter wheat (WW) 56%) and general crops (oil seed rape (OSR) 16%, and potatoes 4%) as example crops for climate change mitigation calculations. At Fen Drayton, temporal changes in management strategies employed at the site were utilised to create hypothetical habitat compositions for the non-intervention and intervention scenarios by scaling up habitat compositions and livestock densities of the oldest and newest compartments, respectively, to the whole study site area [Table 1].

**Table 1:** Habitat areas for Ouse Fen and Fen Drayton under current and alternative land-use scenarios.

	**Ouse Fen (ha)**		**Fen Drayton (ha)**		
**Habitat type**	**Current state (Nature conservation)**	**Agricultual scenario**	**Current state (Mix of treatments)**	**Intervention treatment**	**Non-intervention treatment**
Grassland	58	-	105	88	23
Open water	21	-	164	160	249
Scrub woodland	6	-	27	32	37
Reedbed	69	-	15	32	2
Arable farmland	-	153	-	-	-

Carbon (C) stocks in above- and below-ground biomass and litter were calculated using published literature values for each habitat type [21, 22]. Previous sampling at Ouse Fen indicates soil types at the site are mineral [23]. Therefore, given the proximity of Ouse Fen to Fen Drayton [Fig. 1], soil organic matter (SOM) C stocks at both sites were calculated based on corresponding mineral soil conversion factors [24]. Given the lack of standardisation among published literature uncertainty values, errors in C stocks were estimated as 90% of mean values following standard guidelines [22].

Net CO_2_ and N_2_O fluxes associated with soil and vegetation in grassland areas [25] were used for extensively grazed grassland on mineral soil. Emissions of CH_4_ from livestock were estimated by using per head values for beef cattle grazing temperate grassland [22], and emissions of N_2_O from manure by using values scaled by the proportion of time spent grazing the site each year [22]. Net CO_2_ fluxes from mature deciduous woodland and scrubland areas were estimated from [26] (lower estimate, measured in Oxfordshire, UK) and [27] (upper estimate, modelled). The same estimates were used for mature deciduous woodland and scrubland because of the paucity of published values for scrubland or non-climax woodland. We assumed that the CO_2_ flux of rapidly growing scrub and new woodland (pre-climax communities) would lie somewhere between the minimum and maximum likely sequestration rates of mature woodland stands, given that peak sequestration in tree stands occurs around the period of canopy closure (typically 5–20 Mg CO_2_ ha^-1^ a^‑1^ [27]). Fluxes of N_2_O and CH_4_ from scrubland and woodland areas with unfertilised mineral soils were considered to be insignificant [21, 27]. We derived upper and lower estimates of net fluxes of CO_2_ and CH_4_ from reedbed areas [28, 29], but (in the absence of published data) omitted greenhouse gas fluxes from open water. Emissions of CO_2_ from soil organic matter oxidation were estimated for arable farmland [24]. Direct and indirect (volatilisation and leaching) N_2_O emissions were derived following assuming 160 kg ha^-1^ nitrogen fertiliser addition annually for WW, 155 kg ha^-1^ for OSR and 70 kg ha^-1^ for potatoes [22, 30]. Emissions of CH_4_ from arable farmland were not calculated as these are considered to be negligible from aerobic soil environments[21].

Overall C storage for the current and alternative land use scenarios at each site was calculated as the sum of above- and below-ground biomass, dead wood, litter and SOM per unit area for each type of habitat multiplied by habitat area. We converted net flux of each gas (Mg ha^-1^ a^-1^) into Mg CO_2_ equivalents (CO_2_-eq) ha^-1^ a^-1^, and summed these to give a net global warming potential (over 100 years, GWP_100_) ha^-1^ a^-1^ under each land use [31]. These values are also expressed as a total value of Mg CO_2_-eq a^-1^ for the whole of each site. We used the standard convention of positive values indicating net atmospheric warming, and expressed results as a range based on minimum and maximum GHG emissions values. Values of global climate change mitigation benefits attributable to carbon storage and sequestration were explored using a range of published C prices.

### Livestock Grazing

The RSPB does not currently impose a fee on graziers in return for grazing rights on grassland areas at Ouse Fen. Therefore, the current value of livestock grazing at the site was based on the assumption that graziers would be charged for grazing elsewhere, as confirmed in an interview with a local grazier. This value was derived from the per unit area charge applied currently at the neighbouring Fen Drayton site (below). Under the agriculture scenario no grazing occurs at Ouse Fen.

Grazing at Fen Drayton is charged currently to the grazier for access to a total of 143 ha (70 ha of the study site and an additional 73 ha of adjacent land) for which a charge per unit area was derived. For the intervention scenario, the proportion of land grazed in the newest compartment of the reserve was scaled up to the whole site and the value of grazing calculated by multiplying this area by the current grazing charge per unit area. Under the non-intervention scenario no grazing occurs at Fen Drayton.

### Crop production

Due to sensitivity issues of disclosing financial information of individual farm business, income figures from crop production immediately around Ouse Fen were unavailable. Therefore, we estimated the value of gross farm income from crop production for the agriculture scenario using the regional average cropping ratio as above [20] and regional average farm business figures from the 2010/2011 Farm Business survey [32]. Farm business figures were adjusted to exclude income and expenditure not directly associated with crop production, such as agricultural subsidies received under the European Union Common Agricultural Policy (since these represent an internal transfer of benefit between societal constituents [33]), and rent or interest accrued through land ownership/capital holdings [34].

### Nature-based recreation

Recreational values for each site were determined using individual visitor questionnaires conducted during June/July 2012 (see S1 and S2 Texts for interview questions). Data were collected over seven days at Ouse Fen and 11 days at Fen Drayton and spanned a range of school days and school holidays. Daily visit numbers were also recorded. We did not seek ethical approval for visitor surveys because our questions involved no categorization by race/ethnicity, age, disease/disabilities, religion, sex/gender, sexual orientation, or other socially constructed groupings. Data were collected and analysed anonymously and oral consent was sought for each respondent at the beginning of each survey. Where consent was not granted, interviews were terminated and any collected data were deleted. Written consent was considered to be inappropriate due to the short duration of each interview (typically < 3 mins) and the potential for obtaining written consent to reduce participant numbers substantially and bias results. Annual visit numbers were unavailable for either site. Therefore, we used data from a nearby restored wetland (Wicken Fen, Cambridgeshire, UK) for which daily and annual visit numbers were available. Annual visit numbers to Ouse Fen and Fen Drayton in their current states were estimated on the assumption that the proportion of the entire year’s visits which occurred on our survey days was the same at Ouse Fen, Fen Drayton and Wicken Fen. The annual value of the recreational benefits provided by each site was calculated as the estimated annual number of visits multiplied by mean spend per visit, defined as the sum of the cost per visit of travel to the site and any additional spend in the local area (as estimated from responses to our questionnaires). For international visitors, we calculated the cost of travel based on the distance from their place of residence while in the UK, rather than their home country. Note that in focusing on direct expenditure we did not consider other aspects of recreational value such as consumer surplus (for detailed discussion see [35]).

To estimate the recreational value of the agriculture scenario at Ouse Fen visitors there were asked if they would still visit the site if it had been restored to farmland, and the proportion answering ‘yes’ then multiplied by the annual number of visits to the site in its current state and by the mean spend of this subset of visitors. Visitors to Fen Drayton were asked which compartments of the site (intervention, non-intervention, or a mixture) they preferred. We estimated the proportion of current visits that would occur under each scenario by making ratio-based adjustments to visitor numbers based on relative popularity ratings between different compartments. These figures were used to calculate annual recreational values for the site under different intervention scenarios.

### Fishing

No fishing occurs at Ouse Fen under the current or agriculture scenarios. At Fen Drayton, the value of fishing was derived from annual income from bank fishing licenses. Under the intervention scenario no fishing occurs at Fen Drayton. For the non-intervention scenario, the perimeter of water fished in the oldest compartment of the reserve was scaled up to the whole site and annual value of fishing calculated by multiplying this by the current fishing charge per unit length per unit area.

### Flood-risk mitigation

Though situated in the historical floodplain of the river Great Ouse, Ouse Fen is separated from the river by high flood banks (breached only once in the last century) and instead receives water from de-watering activities in active extraction areas of the site. In times of flood, excess water flows down the river, past Ouse Fen, to be stored in flood detention areas lower in the catchment and therefore the flood risk mitigation benefits provided by Ouse Fen are negligible.

Fen Drayton is also situated in the floodplain area of the river Great Ouse. During periods of high discharge the site acts to retain water and in doing so provides flood mitigation benefits to nearby villages. Monetary values of flood risk mitigation were not calculated because regional-scale value transfer estimates were unavailable, and also because a monetary ecosystem service comparison was not the aim of the study. Instead, we adopted a more tractable approach, assessing simply the physical capacity of the site to mitigate flood risk by calculating the sum of above- and below-ground water storage provided by the site in its current state. Above-ground storage at the site was calculated for each major compartment as the volume between the mean water table and the bank top using a digital elevation model (1 m horizontal resolution, 0.01 m vertical resolution). The volume of soil above the water table in each compartment was also calculated and then multiplied by the drainable porosity of the soil (taken as 0.25 for fine gravel [36]) to derive below-ground storage for each compartment [37]. The mean volume of water stored per unit area for all compartments was multiplied by the area of Fen Drayton to estimate the total water storage capacity in its current state. For the alternative scenarios, mean volumes of water stored per unit area were calculated for the intervention and non-intervention compartments and then scaled across the entire site.

### Restoration and management costs

One-off restoration and annual management costs were calculated for the different states at each site based on current industry figures for restoration costs per unit area of habitat (N. Symes, pers. comm.), annual RSPB management data [18] and agricultural production costs [32]. Farm expenditures included manual labour, interest and rental costs, and also an additional value for unpaid labour (predominantly that of the farmer and spouse) which, although often excluded from reported values, constitutes a genuine cost to agricultural production. Note that these values are presented to place the restoration strategies in a management context, and are not intended to represent a full cost-benefit analysis.

## Results

### Global climate change mitigation

**Fig 2:**
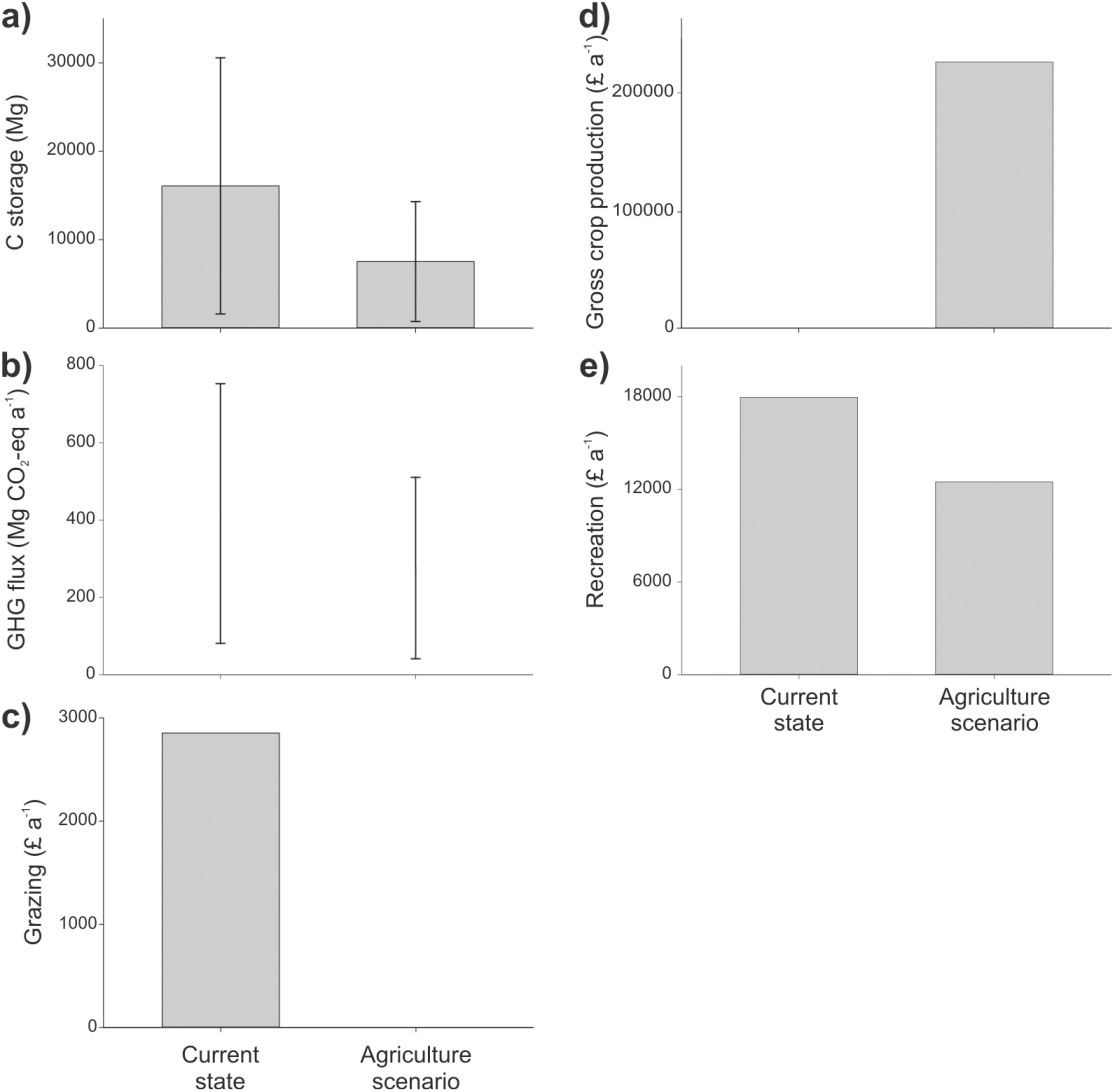
Ecosystem services provided by Ouse Fen under the current (nature conservation) and alternative (agricultural) land use scenarios. Bars for C storage represent mean values and error bars represent upper and lower estimates assuming errors of ±90% mean values.

**Fig 3:**
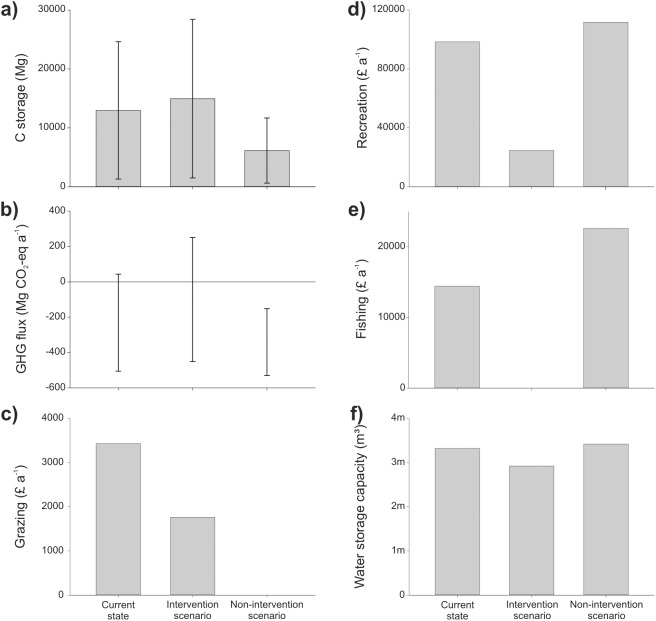
Ecosystem services provided by Fen Drayton under the current management regime and alternative intervention and non-intervention scenarios. Bars for C storage represent mean values and error bars represent upper and lower estimates assuming errors of ±90% mean values.

The total C stock of Ouse Fen in its current nature conservation state is estimated at 16087 ±14478 Mg C, of which more than half is stored in SOM [Table 2; Fig. 2A]. Reedbed habitat represents 70% of total C storage at the site. Under the agricultural scenario, total mean C storage is less than half that of the current state, with almost 90% stored as SOM. Carbon storage at Fen Drayton is estimated to be 12962 ± 11666 Mg C in the current state. This figure is approximately 2000 Mg less than in the intervention scenario, but twice that of the non-intervention scenario [Table 2; Fig. 3A].

**Table 2:** Mean C storage by habitat type at Ouse Fen and Fen Drayton under current and alternative land-use scenarios.

**Site and scenario**	**Habitat type**	**Habitat coverage (%)**	**Carbon storage (Mg)**				
			**AGB**	**BGB**	**Litter**	**SOM**	**Total**
Ouse Fen—Nature conservation (current state)	Grassland	38	63	407	129	3486	4084
	Scrub woodland	4	143	143	13	376	676
	Reedbed	45	4832	651	634	5209	11327
	Open water	13	0	0	0	0	0
	**Total**		**5038**	**1201**	**776**	**9071**	**16087**
Ouse Fen—Agricultural scenario	Cropped land	100	**721**	**153**	**57**	**6596**	**7527**
Fen Drayton—Mix of treatments (current state)	Grassland	34	114	735	233	6304	7386
	Scrub woodland	9	640	640	59	1681	3021
	Reedbed	5	1090	147	143	1175	2556
	Open water	53	0	0	0	0	0
	**Total**		**1844**	**1523**	**436**	**9160**	**12962**
Fen Drayton—Intervention scenario	Grassland	28	95	613	194	5255	6157
	Scrub woodland	10	760	760	70	1994	3583
	Reedbed	10	2228	300	292	2402	5223
	Open water	51	0	0	0	0	0
	**Total**		**3082**	**1673**	**557**	**9651**	**14963**
Fen Drayton—Non-intervention scenario	Grassland	7	25	160	51	1369	1604
	Scrub woodland	12	889	889	82	2332	4192
	Reedbed	1	149	20	20	161	350
	Open water	80	0	0	0	0	0
	**Total**		**1062**	**1068**	**152**	**3862**	**6145**

AGB, BGB and SOM denote above-ground biomass, below-ground biomass, and soil organic matter, respectively.

Net GHG flux at Ouse Fen is 81 to 752 and 41 to 511 Mg CO_2_-eq a^-1^ in the current state and agriculture scenario, respectively [Fig. 2B], and attributable primarily to CH_4_ production from anaerobic reedbed habitats in the current state and N_2_O emissions from mineral fertilisers in the agricultural scenario. Net GHG flux at Fen Drayton is 44 to -506 Mg CO_2_-eq a^-1^ in the current state, 251 to -450 Mg CO_2_-eq a^-1^ in the intervention scenario, and -152 to -531 Mg CO_2_-eq a^-1^ in the non-intervention scenario [Fig. 3B]. Monetary values of C storage [Table 3] and C sequestration [Table 4] vary by one to two orders of magnitude depending on C price.

**Table 3:** Sensitivity analysis of mean C storage valuation.

**Source**	**£ Mg CO**_2_ **-eq** ^-1^	**C storage (£)**				
		**Ouse Fen**		**Fen Drayton**		
		**Currentstate**	**Agriculture scenario**	**Currentstate**	**Intervention scenario**	**Non-intervention scenario**
EU Emission Trading Scheme (Point Carbon, 2011)	3.95	233,497	109,249	188,148	89,189	217,190
US Government (Greenspan Bell and Callan, 2011)	15.26	900,652	421,400	725,731	344,024	837,752
UK Government Central Scenario (DECC, 2013)	3.49	206,044	96,405	166,027	78,703	191,655
UK Government High Scenario (DECC, 2013)	15.57	919,230	430,092	740,701	351,120	855,033
Tol (2010)	21.47	1,267,292	592,945	1,021,165	484,071	1,178,788
Stern et al. (2006)	63.28	3,735,717	1,747,879	3,010,183	1,426,940	3,474,823
Verified Emission Reductions (Peters-Stanley et al., 2011)	4.14	244,164	114,240	196,744	93,264	227,113

Carbon prices were adjusted to 2013 based on IMF (2013) inflation rates. US$ values were converted to UK£ using an exchange rate of $US1.62/UK£.

**Table 4:** Sensitivity analysis of median C sequestration valuation.

**Source**	**£ Mg CO**_2_ **-eq** ^-1^	**C sequestration (£ a** ^-1^ **)**				
		**Ouse Fen**		**Fen Drayton**		
		**Currentstate**	**Agriculture scenario**	**Currentstate**	**Intervention scenario**	**Non-intervention scenario**
EU Emission Trading Scheme (Point Carbon, 2011)	3.95	-1,649	-1,092	915	395	1,350
US Government (Greenspan Bell and Callan, 2011)	15.26	-6,361	-4,210	3,527	1,522	5,208
UK Government Central Scenario (DECC, 2013)	3.49	-1,455	-963	807	348	1,192
UK Government High Scenario (DECC, 2013)	15.57	-6,493	-4,297	3,600	1,553	5,316
Tol (2010)	21.47	-8,951	-5,924	4,963	2,141	7,329
Stern *et al*. (2006)	63.28	-26,386	-17,464	14,631	6,312	21,604
Verified Emission Reductions (Peters-Stanley *et al*., 2011)	4.14	-1,725	-1,141	956	413	1,412

Carbon prices were adjusted to 2013 based on IMF (2013) inflation rates. US$ values were converted to UK£ using an exchange rate of $US1.62/UK£.

### Grazing

The value of grazing at Ouse Fen is estimated to be £2854 a^-1^ based on a standard charge for grazing rights of £49 ha^-1^ a ^-1^. This value is zero in the agricultural scenario [Fig. 2C]. At Fen Drayton, the value of grazing is estimated to be £3433 a^-1^ in the current state of the site, £1763 a^-1^ in the intervention scenario, and zero in the non-intervention scenario [Fig. 3C].

### Crop production

The value of crops at Ouse Fen under the agriculture scenario was estimated from regional average values for gross farm income of £1215 ha^-1^ a^-1^ for cereals and £1928 ha^-1^ a^-1^ for general cropping, yielding a total gross annual income of £226,258 when cropped at the regionally typical ratio of 63:37 cereals to general cropping [Table 5; Fig. 2D].

**Table 5:** Outputs and costs associated with the agricultural restoration scenario at Ouse Fen assuming a regionally-characteristic ratio of 63:37 cereals to general cropping.

** **	**Cereals (**£ **ha** ^-1^ **)**	**General cropping (**£ **ha** ^-1^ **)**	**Agricultural scenario (**£ **ha** ^-1^ **)**	**Total (**£**)**
Total agricultural output (excluding subsidies)	1215	1928	1479	226258
Less: income from miscellaneous activities	-109	-78	-98	-14922
**Outputs** attributable to cultivated goods	1106	1850	1381	211336
Total fixed and variable costs	888	1473	1104	168981
Plus: unpaid labour	86	75	82	12535
Less: net interest and rent	-74	-147	-101	-15455
Less: costs of miscellaneous activities	-68	-57	-64	-9781
**Costs** attributable to cultivated goods	832	1344	1021	156280
**Net income attributable to cultivated goods**	274	506	360	55056

### Nature-based recreation

A total of 23 questionnaires were completed at Ouse Fen (see S1 Dataset for full dataset). Most visitors travelled from the local area by car with a mean travel distance of 16 km [Table 6]. Approximately 4,500 visits were estimated for the site each year in its current state and the annual recreational value of the site was calculated as £17,950. Under the agricultural scenario, annual visit numbers were estimated to be reduced to 3,129 and the annual recreational value of the site was estimated as £12,487 [Fig. 2E].

**Table 6:** Descriptive statistics for visitors to Ouse Fen and Fen Drayton.

		**Ouse Fen**	**Fen Drayton**
Transport (%)	Car	48	51
	Bicycle	13	16
	Walk	30	21
	Bus	0	8
	Other	9	4
Mean number in party	Adults	1.39	1.69
	Children	0.17	0.25
Mean ± SD distance travelled (km)		16.16 ± 13.54	15.82 ± 28.48
Mean ± SD spend per visitor (UK £)		3.99 ± 8.27	4.52 ± 9.54

At Fen Drayton, 216 questionnaires were completed. As for Ouse Fen, the majority of visitors lived locally to the site and travelled by car [Table 6].Total visit numbers to the site in its current state were estimated to be 21,730 a^-1^ with a recreational value of £98,346 a^-1^. There was a significant difference in visitor preferences between intervention types, (χ^2^ = 16.254, df = 2, *p*<0.001), with 21% preferring the intervention compartment (recreational value £55,818 a^-1^), 42% preferring the non-intervention compartment (recreational value £111,636 a^-1^) and 37% preferring the current mix [Fig. 3D].

### Fishing

The value of fishing at Fen Drayton under the current state of the site, based on recorded income from fishing licenses, is £14,433 a^-1^. Fishing is absent under the intervention scenario. The value of fishing under the non-intervention scenario, as calculated by scaling up the perimeter of lakes fished in the oldest compartment across the whole site, was calculated to be £22,613 a^-1^ [Fig. 3E].

### Flood-risk mitigation

In its current state, Fen Drayton has an estimated water storage capacity of 3,323,000 m³. Under the intervention and non-intervention scenarios water storage capacity is 2,924,000 m³ and 3,419,000 m³, respectively [Fig. 3F].

### Restoration and management costs

**Fig 4:**
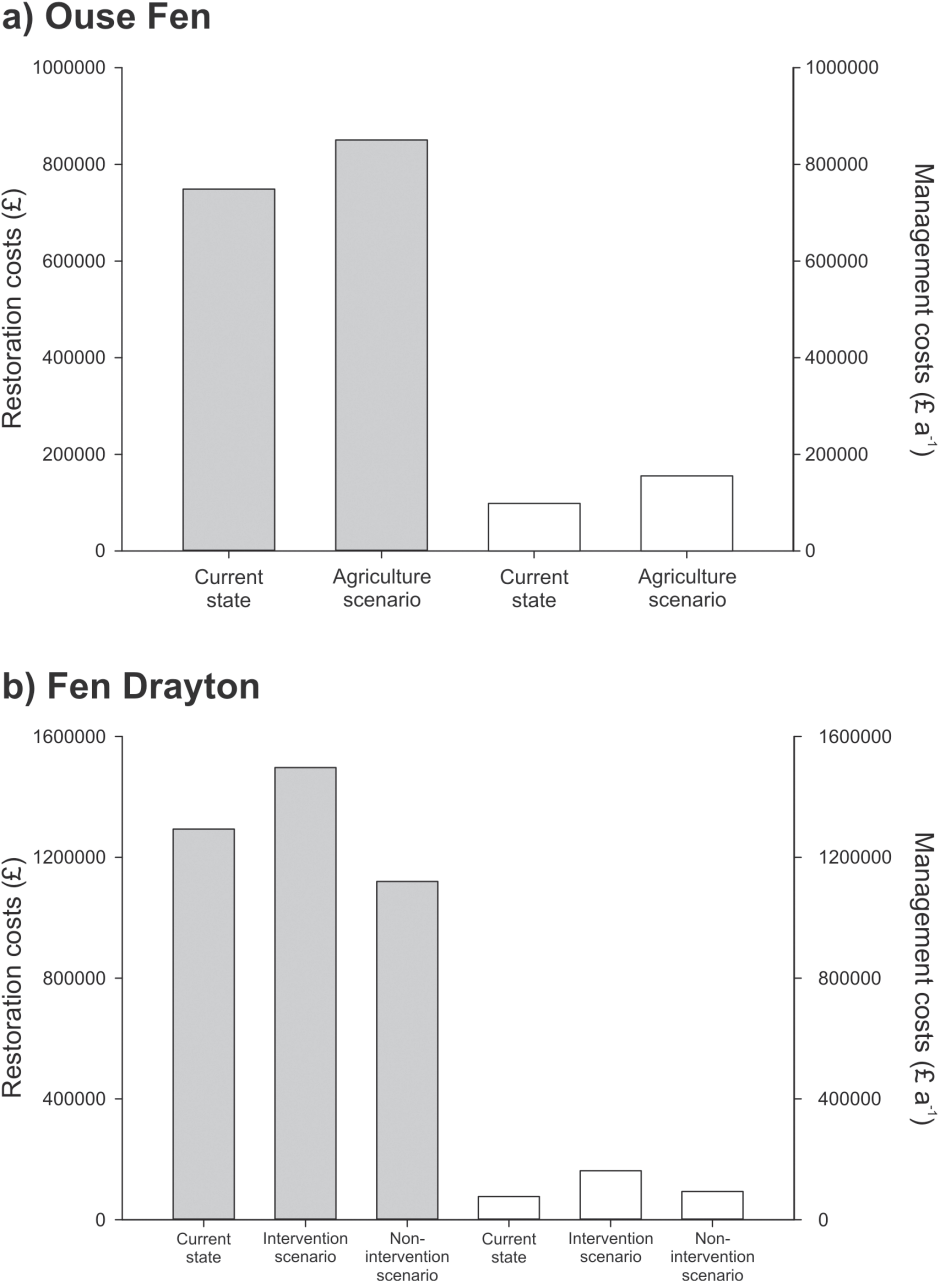
One-off restoration costs (grey bars) and annual management costs (white bars) for a) Ouse Fen under the current and agriculture scenarios, and b) Fen Drayton under the current management regime and alternative intervention and non-intervention scenarios.

At Ouse Fen, one-off restoration costs (approximately £0.8m or £5000 ha^-1^) are similar between the nature reserve and agriculture scenarios [Fig. 4A]. Annual management costs under the agriculture scenario are £1021 ha^-1^, leading to a total net farm income due to arable production of £55,056 a^-1^ [Table 4]. These management costs are considerably more than those incurred as a nature reserve (£650 ha^-1^). At Fen Drayton, restoration costs range from £1.1m to £1.5m (£3600 ha^-1^ to £4800 ha^-1^) between the current state and alternative scenarios [Fig. 4B]. Annual management costs for the current state (£250 ha^-1^) and the non-intervention scenario (£200 ha^-1^) are similar, but costs for the intervention scenario (£520 ha^-1^) are substantially higher.

## Discussion

This study compared the capacity of two common mineral site after-uses—nature conservation and agriculture—to provide ecosystem services, and also explored how the focus of conservation activities mediates the delivery of ecosystem services. The following section examines these topics in turn and subsequently contextualises the results of the study by comparing ecosystem services provision and considering restoration and ongoing site management costs across different management scenarios.

### Ecosystem services provided by nature conservation compared with agriculture

Our results demonstrate that the nature conservation management strategy employed at Ouse Fen provides more than double the mean C storage than would occur under an alternative agricultural land use. This is due largely to the considerable quantity of AGB and SOM stored in reedbed and SOM stored in grassland habitats [21]. The value of this stored C can be considerable, depending on the choice of published C price used, with higher values arising from C prices incorporating social costs and lower values from those reflecting market-based values. Higher C prices may lead to C storage and sequestration becoming a dominant feature in ecosystem service valuations, with the potential for tradeoffs to arise between maximising monetary values and maintaining social equity. With respect to ongoing C fluxes, the nature conservation strategy at Ouse Fen is associated with some CO_2_ sequestration from the atmosphere, yet the concurrent emission of CH_4_ with high GWP_100_ from livestock and reedbeds suggests the site is currently a net emitter of GHGs. Similarly, the agricultural scenario at Ouse Fen is also a net emitter of GHGs, primarily caused by high N_2_O emissions associated with the application of fertilisers. While off-site impacts are not covered in this study, it is notable that the fertiliser production process is energy intensive and generates substantial GHG emissions [38]. Given the relatively wide range of published emission factors employed in this study, our estimates suggest the GWP_100_ of Ouse Fen is comparable under both nature conservation and agricultural management scenarios.

**Fig 5:**
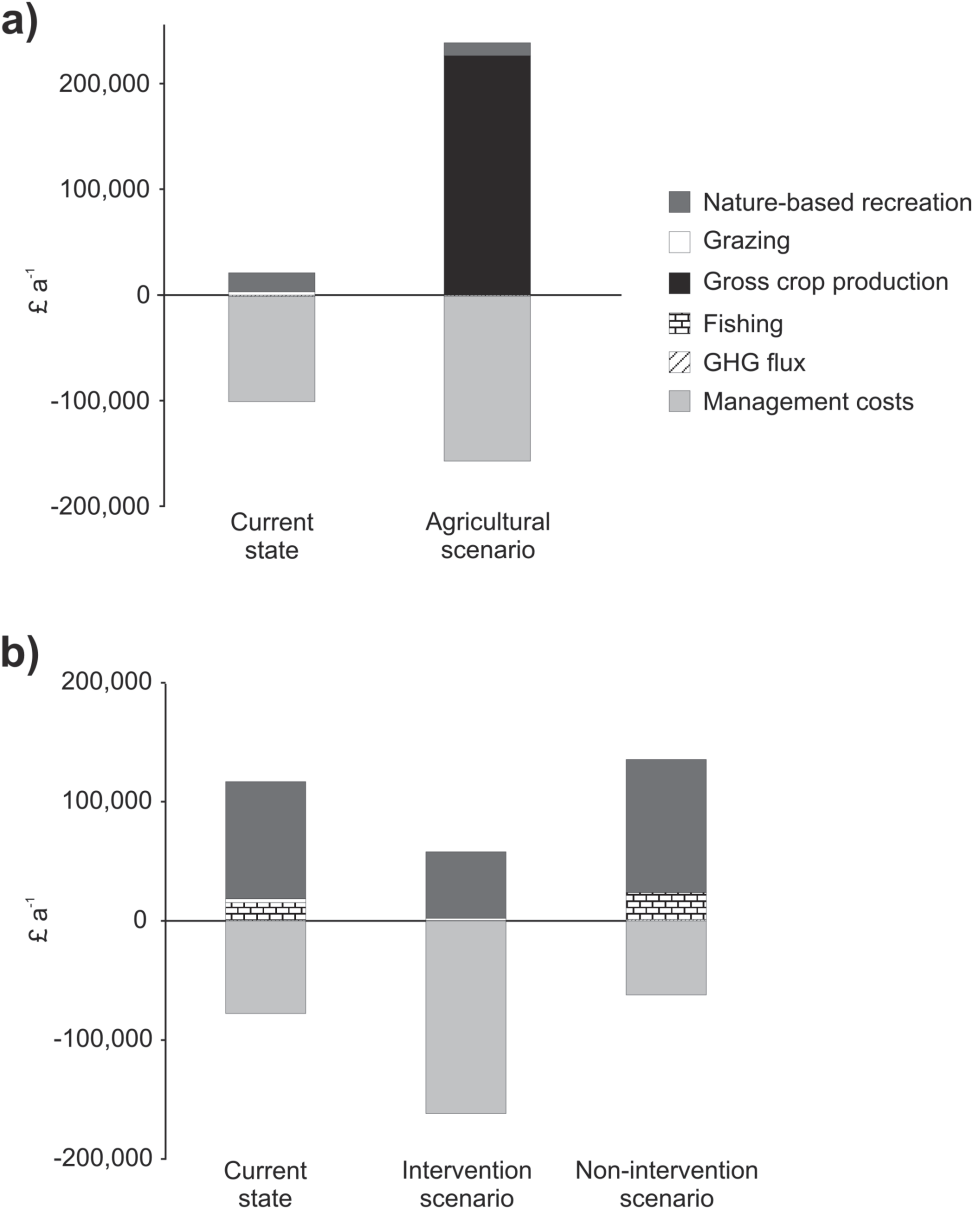
Summary of ecosystem services provision and management costs expressed in £ a^-1^ under different restoration scenarios for a) Ouse Fen and b) Fen Drayton. GHG flux values are based on UK Government central scenario figures. Note that flood risk mitigation values are absent because these are not expressed as monetary values. See text for abbreviations.

In contrast to the results of a similar recent study examining ecosystem services provision at a restored wetland site [34], we found no clear net benefit between either the nature conservation or agricultural management strategies at Ouse Fen. The value of crop production under the agriculture scenario was higher than that of any other service for which monetary values were assigned, but annual management costs associated with this scenario were also higher than those associated with the nature conservation restoration state. Furthermore, increased supply of this provisioning ecosystem service was linked to a trade-off in other regulating and cultural services considered in this study (Fig. 5A). Similar associations between increased agricultural production and declines in other ecosystem services have been reported elsewhere [39, 40], although such patterns are not universal [41]. Provision of some services are habitat area-based, and the provision of one may preclude another (e.g. if all land is used for growing crops then none can be used for grazing), while in other cases trade-offs are not inherent (e.g. a site under crop production could conceivably receive as many visitors one under natural habitats). In fact, almost 70% of sampled visitors to Ouse Fen indicated that they would still visit the site if it had been restored to an agricultural after-use. Although cropland is rarely well-integrated with the provision of recreational space [42], agricultural landscapes in the UK are often interwoven with permissive footpaths. Given that Ouse Fen has only recently been restored, and does not yet have the level of access that might normally be present in a UK nature reserve, it is possible that many visitors use existing historical footpaths for recreation and would still do so if the site had not been restored to a nature reserve.

### Ecosystem services provided by species-focused restoration compared to recreation or mixed-use restoration

The second objective of the study was to understand how the degree of species-focused intervention in nature conservation activities affects ecosystem service provision. Understanding this association is potentially important for site managers given the high management costs that can be associated with implementing nature conservation activities [14]. Our results indicate that ecosystem service delivery at Fen Drayton is influenced to some extent by species-focused interventions, but that this relationship varies on a service-specific basis across the site.

Intervention management areas at Fen Drayton, which are focused on targeted restoration and management actions to support target species of conservation concern, are important providers of carbon storage and livestock grazing, reflecting nature conservation activities that are designed to foster the creation of priority habitat areas. Here, management is centred on encouraging the growth of reedbeds with high volumes of organic matter. In terms of both restoration and ongoing management costs, such activities are relatively expensive in comparison to the non-intervention scenario. Our results indicate that at present Fen Drayton has a climate cooling negative GWP_100_, and would do so under the intervention scenario. Intervention areas are important for the provision of grazing where cattle are used to manage the height of grassland swards, yet livestock also contribute to CH_4_ emissions from the site. Moreover, the current state of the site delivers greater grazing services than it would under either of the two hypothetical scenarios (as it more contains more area under grassland).

Our results indicate that the provision of other ecosystem services, specifically carbon sequestration, fishing and flood risk mitigation, is higher in non-intervention areas at Fen Drayton (Fig. 5B). This is due predominantly to the historical lack of site re-profiling following gravel extraction, which has resulted in the continued presence of steep-banked compartments with large areas of open water in the older areas of the site. Steep banks inhibit the growth of marginal reedbed vegetation [43], but increased areas of open water facilitate improve public fishing opportunities. Additionally, these steep-banked compartments have greater water storage capacity than those with more gentle profiles, and so have increased potential to contribute to reducing the risk of flooding to downstream communities. Given a median annual flood discharge for the Great Ouse river of 85.3 m³ s^-1^ [44] there would be a relatively small time difference of 96 minutes for floodwaters to fill the site between the intervention (11.1 h) and non-intervention (9.5 h) scenarios, suggesting that the primary beneficiaries of increased flood risk mitigation provided by the site under the non-intervention scenario would most likely be to local downstream communities during less extreme flood events. The recreational value of this area of the site was also higher in the non-intervention area than the intervention area and the site in its current state. Given that trails around both areas mean that their accessibility is broadly equal, we suggest that the higher value of the non-intervention area may be linked to the dominance of open water that is considered among the most attractive of landscape elements [45]. However, approximately 40% of all visitors to Fen Drayton preferred the mix of habitats that exists currently at the site, supporting previous studies that have also reported visitor preference for heterogeneity among landscape elements [46, 47].

### Values and limitations of a rapid ecosystem services assessment

This study provided a rapid site-scale assessment of ecosystem services and so has limitations compared to a more comprehensive appraisal. For example, there are other ecosystem services provided by the study sites that were not assessed here because of technical difficulties (e.g. in quantifying existence values) or the risk of double counting (e.g. in estimating the value of pollination to surrounding arable fields). High uncertainty in published values of C storage and GHG fluxes associated with different habitat types underline the potential errors associated with benefit-transfer approaches and the importance of collecting primary site-specific data where possible [48]. Our estimates of recreational and fishing value focus solely on the more tractable elements of direct expenditure incurred by on-site visitors and do not consider consumer surplus (i.e. estimates should be interpreted as the minimum visitors were willing to spend) or the views of people who chose not to visit the sites; and (at Ouse Fen) are limited by a relatively small sample size. Additionally, the study affords only an instantaneous ‘snapshot’ of ecosystem service delivery, rather than considering how service provision may change over time, or how supply is related to demand (e.g. [49]).

However, despite these limitations we suggest our results provide useful indications, based on well-established methods, of the capacity of each site to provide benefits to society. The use of biophysical ecosystem service indicators where possible is advantageous because these are independent of market fluctuations and are more closely related to the ecosystem functions that underpin service delivery [50]. Finally, the approach employed by this study is aimed to be accessible to all stakeholders, and its participatory nature means it can be used to explore the wider consequences of nature conservation and promote discussion between different ecosystem service beneficiaries.

## Conclusion

Ouse Fen and Fen Drayton illustrate some of the different restoration strategies that are possible at post-mineral extraction sites, and the provision of ecosystem services associated with them. Our results show that Ouse Fen as a nature reserve provides more recreational benefits than it would as agricultural land, while at Fen Drayton, the recreation benefits of the site are highest in its current state where different areas of the site have been subject to different levels of intervention, suggesting that a heterogeneous site appeals to more users. Greenhouse gas fluxes and carbon stocks are largely determined by the habitats and their management provided under the various scenarios at both sites. Nature reserve and intervention management provide greater carbon stocks, but none of the options provide especially high levels of sequestration, because both agricultural activities and wetlands are net GHG-emitters. At both sites, the benefits associated with agriculture and grazing are provided in proportion to how compatible these activities are with the restoration scenario.

The implications of changes in ecosystem service provision associated with different restoration strategies should be considered in the context of the beneficiaries, who are largely local (with the exception of the global beneficiaries of climate change mitigation). Under the agricultural scenario at Ouse Fen, local recreational benefits are reduced, and financial benefits of agricultural production are more concentrated (although the provision of food to the wider public is also a consideration). At Fen Drayton, ecosystem service benefits are provided to people who may not visit the site, but who benefit from somewhat increased flood protection, while the different restoration scenarios are likely to appeal to different site visitors.

For conservation managers and restoration advisors, our results highlight the wider socio-economic benefits provided by these restored mineral sites and the dynamics that occur between multiple ecosystem services under different types of restoration and management regime. Because differences in service provision under different restoration scenarios have most impact on local beneficiaries, future site restoration schemes may benefit from adopting a localised (as well as site-specific) planning approach. Nonetheless, we suggest decisions regarding mineral site restoration should not necessarily be based only on utilitarian grounds (cf. [51]). For example, the potential marginal benefits to consumers of crop production from Ouse Fen represent only 0.002% of the national output [52], whereas the site in its current state supports approximately 2.5% of the total bittern population in the UK (S. Wotton, 2014, pers. comm.). Therefore, while improved recognition of ecosystem service delivery may help to inform the restoration of mineral extraction sites, this information must be considered by decision-makers alongside legislative requirements, non-use values and more traditional conservation arguments to develop future restoration schemes that benefit both biodiversity and people.

## Supporting Information

S1 Dataset Recreational survey data from questionnaires at Ouse Fen and Fen Drayton. (DOCX)

S1 Text Interview questions for visitors at Ouse Fen. (DOCX)

S2 Text Interview questions for visitors at Fen Drayton. (DOCX)
