# Supplementary material for: Rapid Assessment of Ecosystem Services Provided by Two Mineral Extraction Sites Restored for Nature Conservation in an Agricultural Landscape in Eastern England
Source: PLoS One. 2015 Apr 20;10(4):e0121010. doi: 10.1371/journal.pone.0121010 (PMC4404093; doi:10.1371/journal.pone.0121010)
Supplement: S1 Dataset — (DOCX) [file pone.0121010.s001.docx]

**Supporting information**

**S3 Dataset. Recreational survey data from questionnaires at Ouse Fen and Fen Drayton.**

A complete dataset of visitor questionnaire data can be accessed using the following URL:

http://dx.doi.org/10.6084/m9.figshare.1288936
