# Supplementary material for: Rapid Assessment of Ecosystem Services Provided by Two Mineral Extraction Sites Restored for Nature Conservation in an Agricultural Landscape in Eastern England
Source: PLoS One. 2015 Apr 20;10(4):e0121010. doi: 10.1371/journal.pone.0121010 (PMC4404093; doi:10.1371/journal.pone.0121010)
Supplement: S1 Text — (DOCX) [file pone.0121010.s002.docx]

**Supporting information**

**S1 Text. Interview questions for visitors at Ouse Fen.**

Interview date:

Time/Location/Weather:

Mode of Transport: bicycle/boat/car/horse/walk

1. When was the first time you visited the site and how often do you come? ______________________________________

2. If applicable, how many persons in the travel group?

Adults __________ Children __________

3. Where are you from? Answer: __________

NB. For visitors who are on holiday/travelling,

(a) origin Town/city: __________

(b) Duration of stay in the UK: __________days

(c) Type of accommodation: __________

4. Have you spent/do you plan to spend money during this trip? Yes □ No □

NB. This include meals and drinks, souvenirs and other purchase during this trip

If yes, how much?­­­­___________________ (estimate spent as a group, if applicable)

5. What are your main reasons for visiting? (NB. If more than one reason, rank them; 1- most important)

Appreciating/viewing nature and/or wildlife □

Exercise, sports or hobbies (include horse-riding, dog walking) □

Time with family or friends □

Others­ _____________________________ □

6. Please indicate what percentage of your reason for visiting is for appreciating/viewing nature and/or wildlife. (NB. Do not ask this question if "Appreciating/viewing nature and/or wildlife" is not one of the reasons for visiting.)

Answer: __________% or 'Don't know' □

7. Would you visit Ouse Fen if it had remained as farmland, rather than being restored to wetland?

Answer: Yes □ No □ 'Don't know' □

8. If yes, would you visit Ouse Fen as often?
