# Supplementary material for: Rapid Assessment of Ecosystem Services Provided by Two Mineral Extraction Sites Restored for Nature Conservation in an Agricultural Landscape in Eastern England
Source: PLoS One. 2015 Apr 20;10(4):e0121010. doi: 10.1371/journal.pone.0121010 (PMC4404093; doi:10.1371/journal.pone.0121010)
Supplement: S2 Text — (DOCX) [file pone.0121010.s003.docx]

**Supporting information**

**S2 Text. Interview questions for visitors at Fen Drayton.**

Interview date:

Time/Location/Weather:

Mode of Transport: bicycle/car/horse/walk

1. When was the first time you visited the site and how often do you come? ______________________________

2. If applicable, how many persons in the travel group?

Adults __________ Children __________

3. Where have you come from today? ____________________________

4. Have you spent/do you plan to spend money during this trip? Yes □ No □

NB. This includes meals and drinks, souvenirs and other purchase during this trip. International visitors should include air/road travel costs, accommodation, local transport and tour costs.

If yes, how much?­­­­___________________ (estimate spent as a group, if applicable)

5. From where did you enter the reserve today? (Show map) ________________________

6. Which parts of the reserve do you prefer to visit? And why?

The northern loop around Drayton Lagoon and/or Ferry Lagoon (all lakes are more open with large expanses of water and/or grassland) □

The southern loop around Elney Lake and/or Oxholme lake (most lakes are more enclosed with a mixture of habitats) □

Both □

Just along the busway and back. □

7. What are your main reasons for visiting? (NB. If more than one reason, rank them; 1- most important)

Appreciating/viewing nature and/or wildlife □

Exercise, sports or hobbies (include horse-riding, dog walking) □

Time with family or friends □

Others­ ______________________________ □

8. Please indicate what percentage of your reason for visiting is for appreciating/viewing nature and/or wildlife. (NB. Do not ask this question if "Appreciating/viewing nature and/or wildlife" is not one of the reasons for visiting.)

Answer: __________% or 'Don't know' □

9. Would you still come if there were no RSPB wardens, shelters and interpretation boards?

Answer: Yes □ No □ Not as often □
